# LPMP: A Bio-Inspired Model for Visual Localization in Challenging Environments

**DOI:** 10.3389/frobt.2021.703811

**Published:** 2022-02-04

**Authors:** Sylvain Colomer, Nicolas Cuperlier, Guillaume Bresson, Philippe Gaussier, Olivier Romain

**Affiliations:** ^1^ Institut de Recherche Vedecom, Versailles, France; ^2^ Laboratoire ETIS UMR8051, CY Cergy Paris Université, ENSEA, CNRS, Cergy, France

**Keywords:** visual place recognition (VPR), bio-inspired robotics, hippocampus, place cells, neurocybernetics, autonomous vehicle (AV), brain-inspired navigation

## Abstract

Autonomous vehicles require precise and reliable self-localization to cope with dynamic environments. The field of visual place recognition (VPR) aims to solve this challenge by relying on the visual modality to recognize a place despite changes in the appearance of the perceived visual scene. In this paper, we propose to tackle the VPR problem following a neuro-cybernetic approach. To this end, the Log-Polar Max-Pi (LPMP) model is introduced. This bio-inspired neural network allows building a neural representation of the environment *via* an unsupervised one-shot learning. Inspired by the spatial cognition of mammals, visual information in the LPMP model are processed through two distinct pathways: a “what” pathway that extracts and learns the local visual signatures (landmarks) of a visual scene and a “where” pathway that computes their azimuth. These two pieces of information are then merged to build a visuospatial code that is characteristic of the place where the visual scene was perceived. Three main contributions are presented in this article: 1) the LPMP model is studied and compared with NetVLAD and CoHog, two state-of-the-art VPR models; 2) a test benchmark for the evaluation of VPR models according to the type of environment traveled is proposed based on the *Oxford car dataset*; and 3) the impact of the use of a novel detector leading to an uneven paving of an environment is evaluated in terms of the localization performance and compared to a regular paving. Our experiments show that the LPMP model can achieve comparable or better localization performance than NetVLAD and CoHog.

## 1 Introduction

The performance of robotic localization systems depends on their ability to continuously build a stable and accurate representation of their environment (Yurtsever et al., 2019). However, building such a representation remains a challenge for self-driving cars, which must face large and dynamic environments since they are intended to be deployed over long periods in environments of several tens of kilometers. Even on the scale of a single day, changing conditions such as variations in light, the transient presence of vehicles or pedestrians, and unpredictable changes in the urban landscape (road works) particularly affect the perception of space (Zaffar et al., 2019). Solving these problems is essential for the deployment of autonomous vehicles.

Among the different available sensors to perform robotic localization, the use of vision is gaining more and more interest since cameras are rich, passive, and inexpensive sensors (Van Brummelen et al., 2018). The domain of visual place recognition (VPR), which aims to characterize a place from visual information, has given rise to numerous research works these last years (Yurtsever et al., 2019). These systems operate mainly by processing information acquired from a monocular camera in order to self-localize in an environment. The current location is found by searching, among the places already visited, the one with the appearance closest to the current image.

With the applications of VPR being not only limited to the field of navigation, several communities have proposed models, resulting in a very rich landscape of models (Chen et al., 2017b; Zaffar et al., 2020a). Thus, numerous approaches have been proposed, first based on hand-crafted local or global features and more recently relying on deep networks (Arandjelović et al., 2016; Zhang et al., 2021). These approaches offer different balances between computing cost and performance, which defines a horizon of possible use cases. For example, convolutional neural network (CNN) models are among the best-performing networks in the state of the art and allow obtaining high localization scores. They are, however, quite resource-consuming and need to be learned on large datasets, which still limits the use cases to which they can be applied in the field of robotic navigation (Zhang et al., 2021).

These last decades, a few works proposed addressing the VPR problem *via* bio-inspired architectures based on the neural mechanisms underpinning the spatial cognition in animals (Zeno et al., 2016). Studies of animal spatial cognition can indeed serve as a blueprint to design innovative models aiming at endowing artificial systems with capabilities akin to the biological ones. From the large literature on the spatial cognition of mammals, these works propose models recreating the interactions of brain structures where spatially tuned neurons (i.e., place cells) are found (Grieves and Jeffery, 2017). Thus, they offer architectures situated at the interface between robotics and the field of the computational neuroscience (neurorobotics). The resulting neural architectures led to solutions for robot navigation (both indoor and outdoor), which may represent alternatives to “classic robotics” ones. Their applications to localization problems have thus demonstrated that they could be efficient, offering new robustness and adaptive properties (Gaussier et al., 1997; Milford et al., 2004; Cuperlier, 2007; Ball et al., 2013).

In this paper, we studied the integration of a bio-inspired localization model called LPMP (Log-Polar Max-Pi) on robotic localization issues (Espada et al., 2019). Our goal was to determine the key elements of the model allowing, despite its simplicity, to provide competitive localization results. Our contributions are the following:

• We performed a rigorous evaluation of LPMP under challenging conditions and compared it with NetVLAD (Arandjelović et al., 2016) and CoHog (Zaffar et al., 2020b), two VPR solutions among the most efficient of the state of the art.
• We studied the different sequences available on the *Oxford car dataset* in order to build a testing benchmark, allowing to compare the performance of the models between different key environments.
• We evaluated two ways of constructing an environment representation: an automatic mechanism (called the *vigilance system*), which triggers the registering of a new place if this place is not sufficiently recognized, and a static method, which consists of recording an image every *x* meter.

The rest of this article is divided as follows: firstly, a brief review of the VPR field and of visual localization in biological systems is provided. Subsequently, the general operation of the LPMP model is introduced. Finally, the experiments performed and the results obtained are presented and then discussed.

## 2 VPR and Large-Scale Localization

### 2.1 General Definition

**FIGURE 1:**
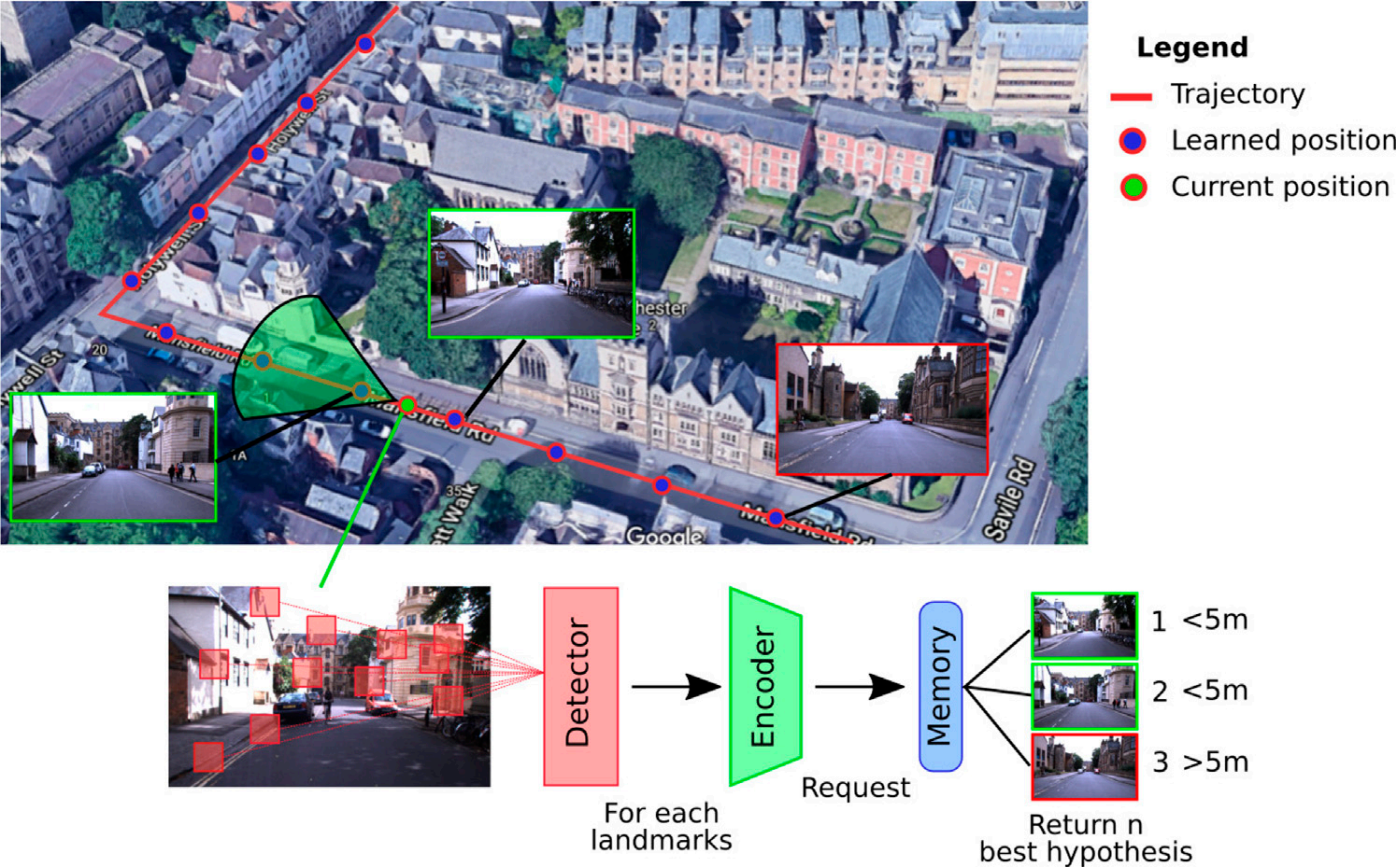
Illustration of a classic visual place recognition (VPR) operation. A VPR system can be decomposed into three functional blocks: 1) a detector that selects the significant information of the acquired image; 2) an encoder that compresses the useful information; and 3), a memory that contains the places in a memory format suitable for the query system. The final code is used to determine from the current image of the vehicle in which place it is located by extrapolating its position from the responses of the system.

Coming from the robotics community, the VPR problem is traditionally posed as a research task in a tagged image database (Figure 1). In general, a request in the form of an image is sent to the system, which must determine the most likely places to which it belongs. VPR models are often associated with the problem of simultaneous localization and mapping (SLAM), which requires regularly determining whether a place has already been visited (problem of loop closure detection) (Bresson et al., 2017).

Whether it is to respond to a request or to build a map of an environment, a VPR system always follows a similar pathway: it starts with an image acquisition, followed by some image processing that allows building a representation that characterizes the current location (Arandjelović et al., 2016; Zhang et al., 2021). This general process can thus be summarized in a functional architecture by three blocks, as described in the Figure 1 (Chen et al., 2017b). Depending on the system, the distinction between these three blocks can be quite blurry, especially with models that encode the image entirely. However, even if the image processing is global, these systems still have to carry out a form of information selection followed by its encoding.

Being a well-posed problem, the method for evaluating the performance of VPR models is fairly conventional (Zaffar et al., 2020a). Firstly, the target VPR system encodes a particular sequence of images (learning dataset), for which the ground truth is known and recorded. Secondly, several images (test dataset) are presented to the system, which returns the closest known images called hypotheses. To establish the performance of the model, it is then sufficient to qualify the hypotheses of the system according to their distance to the current position of the vehicle.

### 2.2 Brief Review of VPR

Due to its multidisciplinary nature, the field of VPR has been studied by several communities and used in a wide variety of applications: in machine vision (Torii et al., 2013; Sermanet et al., 2014; Zaffar et al., 2020b), in databases (Park et al., 2010; Arandjelović et al., 2016), and in robotics (Bresson et al., 2017; Siam and Zhang, 2017; Garg et al., 2018). A short overview of the different VPR categories is presented in the following.

#### 2.2.1 Local Handcrafted Feature Models

The oldest approaches were based on handcrafted descriptors computed from local features (Schmidt and Kraft, 2015), such as SIFT (scale-invariant feature transform) (Lowe, 1999), SURF (speeded up robust features) (Bay et al., 2008), DBow (bags of binary words) (Galvez-López and Tardos, 2012), and ORB (oriented FAST and rotated BRIEF) (Rublee et al., 2011). These approaches have the disadvantage of being very dependent on the quality of the detector used, often quite sensitive to variations in brightness or to the proportion of irrelevant elements (pedestrians, bicycles, or vehicles) on an image.

#### 2.2.2 Global Handcrafted Feature Models

Global handcrafted approaches process the entire image to characterize it, without going through a detection phase. These approaches are often more resistant to changes in illumination and to the presence of small irrelevant elements than are local methods, but they are more sensitive to the change of point of view or weather variations (Zaffar et al., 2019). Among the most efficient global features from the state of the art, we can cite GIST (Oliva and Torralba, 2006) or CoHog (Zaffar et al., 2020b), which offer very efficient and light image characterization methods.

#### 2.2.3 Learning Approaches

With the development of computing power, many fields have seen the emergence of new models based on deep learning techniques such as CNNs or adversarial models (Chen et al., 2017c; Robert et al., 2018; Zaffar et al., 2020b). The first work carried out in VPR was to determine whether using pre-trained networks could allow obtaining acceptable localization performance by relying on the capacity of CNNs to find efficient features to characterize an image (Sermanet et al., 2014). Now, several models offer complete chains to solve the VPR problem, giving very good results in terms of performance in difficult localization conditions.

Among these approaches, we can cite NetVLAD (Arandjelović et al., 2016), HybridNet (Chen et al., 2017c), and AMOSNet (Robert et al., 2018), which offer the best results in the state of the art (Zaffar et al., 2020a). However, the downsides of such methods are the computational cost (whether in learning or in use), their need for large learning datasets, and their lack of explainability, which are important criteria when considering autonomous driving.

### 2.3 Large-Scale Deployment Constraints

The deployment of a VPR system on large scales of distance and time presupposes finding solutions to several issues related to the use of a visual sensor. A brief summary of the main constraints on an increasing timescale is given in this section:

- *Sensitivity to viewpoint changes:* On the same trajectory, the point of view can change drastically, especially when turning or at high speeds. This problem is one of the most critical for a localization system.
- *Robustness to dynamic environments:* Over a very short time interval, the appearance of places in an environment can vary due to human activity. VPR systems must be able to continue to recognize a location despite the presence of irrelevant elements such as pedestrians, vehicles, or roadworks.
- *Robustness to light conditions:* Throughout a day, the light conditions change and can modify the colorimetry of a place, cast shadows differently, etc. The glare of a visual sensor thus causes a strong loss of visibility, leading to a decrease in localization performance.

## 3 Visual Localization in Biological Systems

If the deployment of VPR systems on a large scale remains a difficult challenge, biological systems such as mammals show us that it is nevertheless possible to find light and efficient solutions. Many species are indeed able to travel hundreds of kilometers to ensure their survival, especially during animal migrations (Tsoar et al., 2011). Based on these observations, several works have started to model the cognitive processes underlying animal navigation in order to propose original solutions for the navigation of artificial systems (Milford et al., 2004; Cuperlier, 2007; Chen et al., 2017a; Espada et al., 2019; Ju and Gaussier, 2020). This section presents some of the main mechanisms involved in the spatial cognition of mammals.

### 3.1 Hippocampal Pathway

To perform localization tasks, animals rely essentially on two sources of information (Whishaw et al., 2001; Giovannangeli et al., 2006a): *allothetic* information, which are external signals such as visual cues, and *idiothetic* information, coming from internal sensors, sensible to self-movement information such as the vestibular system or proprioception. These pieces of information are retrieved in mammals in the different neocortices of the brains that are involved in the extraction of one modality (Yoder et al., 2011).

Then, these pieces of information are processed separately in two pathways (Goodale and Milner, 1992; Saleem, 2020): the ventral stream or *what* pathway involved in the recognition of objects and the dorsal stream or *where* pathway specialized in the processing of spatial information. Lastly, they reach respectively the perirhinal cortex (LaChance et al., 2019) and the parahippocampal cortex (Aminoff et al., 2013) to be merged in the hippocampal system (HS), known to play a key role in spatial memory (Zola-Morgan et al., 1989; Eichenbaum, 2017). The brain seems to process the what visual information in a very similar way to that followed by VPR systems, where the first stages of the visual system behave similarly to visual keypoint detectors and encoders. On the contrary, the where information coding the spatial configuration of the point of interest (PoI) *via* their azimuth angles is usually missing from a classic VPR system.

**FIGURE 2:**
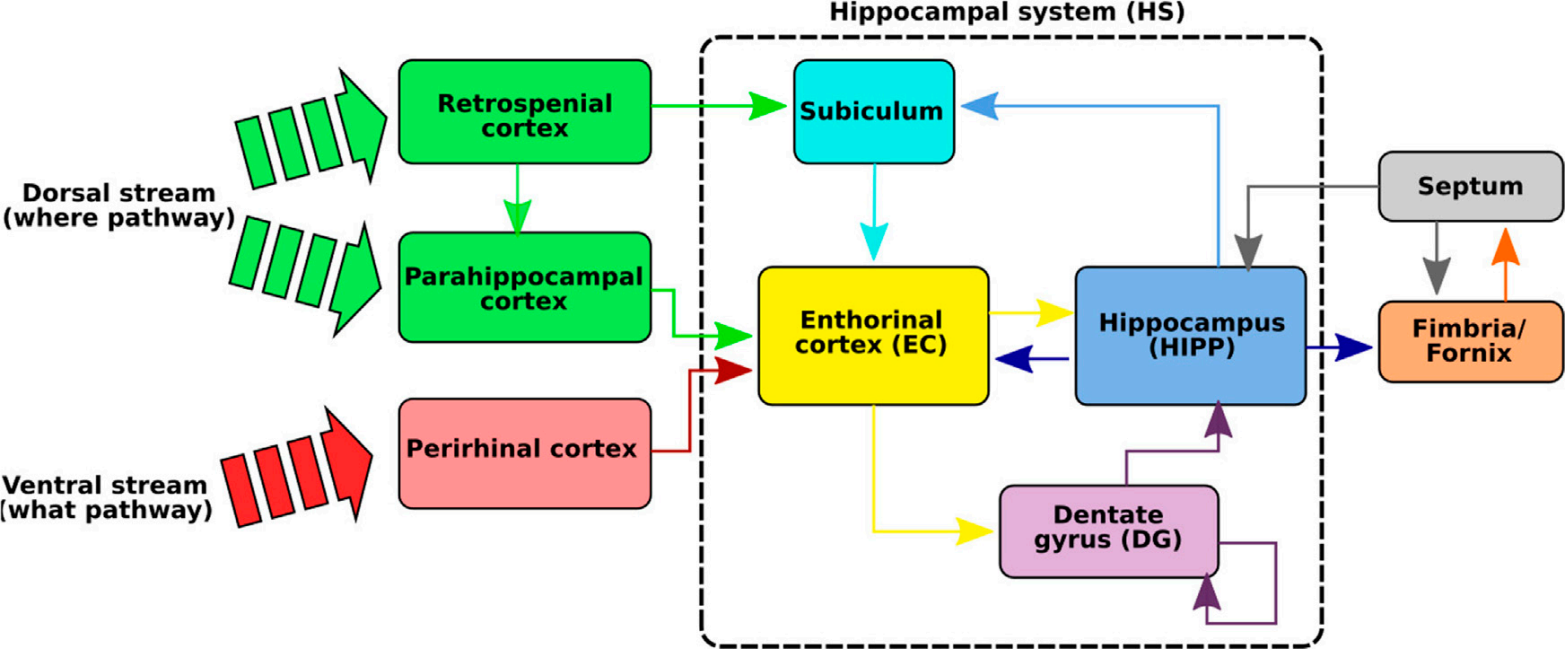
Illustration of the hippocampal system (HS). The functional path of spatial memory begins in the perirhinal and parahippocampal cortices that filter and encode the idiothetic and allothetic information from the neocortices. These two pathways are merged in the entorhinal cortex before reaching the hippocampus, either directly or *via* the dentate gyrus. Several neurons, sensitive to spatial information, have been identified in the HS: *place cells* sensitive to particular positions, *spatial view cells* responding to points of view, and *head direction cells* tuned to specific head directions. A particular loop has also been identified between the hippocampus and the septum (septo-hippocampal loop) and seems to play an important role in the learning of new events in the HS *via* cholinergic modulations in the HS (Hasselmo, 2006).

The HS (Figure 2), composed of the hippocampus proper (HIPP), the dentate gyrus (DG), the enthorinal cortex (EC), and the subiculum (SUB), is one of the brain regions that has been the most studied in neurobiology due to its essential role in spatial cognition (Moser et al., 2015) and in human episodic memory (Lisman et al., 2017). Even if the exact functioning of this system is still not perfectly known, several striking discoveries have been made that allowed us to puzzle out the processes involved in spatial cognition (O’Keefe and Dostrovsky, 1971; Taube et al., 1990; Moser et al., 2015). These studies, mainly conducted on rats and monkeys, have revealed that the HS is made up of a wide variety of spatial neurons forming the neuronal basis of spatial navigation (Robertson et al., 1998; Rolls and Wirth, 2018).

### 3.2 Spatial Map Structure

One of the most important questions in this field remains to determine the role played by the different kinds of spatial neurons found in the HS and how they interact to give rise to a robust and accurate map of the environment supporting the navigation behavior.

- Place cells: The first neurons measured in the HIPP, and surely the most famous ones, are the place cells (PCs). Discovered in 1971 by O’Keefe and Dostrovsky (1971), these neurons encode precise locations in an environment and its surroundings by firing maximally when located in the encoded place. The space encoded by the activation of the neuron, called a place field, shows interesting dynamic and robustness properties (Moser et al., 2015). Initially located in the HIPP, place cells are also found in the DG and the superficial EC (O’Mara, 1995).
- Spatial view cells: These neurons discovered in the HIPP of primates (Robertson et al., 1998; Rolls and Wirth, 2018) are sensitive to a specific point of view (see Figure 2). Thus, unlike place cells, these neurons respond only when the animal is in a specific part of the environment with a given orientation.
- Head direction cells: Discovered in 1984 by Taube et al. (1990), Taube (2003), and Butler et al. (2017), head direction (HD) cells encode the absolute orientation of the animal’s head independently of the position. Their activity can therefore be seen as a compass. First discovered in the SUB, these neurons can be found in different parts of the brain, notably in the retrosplenial cortex (Jacob et al., 2017).

## 4 LPMP Model

This section is dedicated to the full explanation of the proposed model. It begins with an overview of each element composing the model and its interactions, followed by a detailed explanation of each block.

### 4.1 Complete Overview

In this paper, we propose using the neuro-cybernetic model “Log-Polar Max-Pi” (LPMP) (Giovannangeli et al., 2006a; Cuperlier, 2007) to resolve VPR on autonomous vehicles in challenging environments. This model, using local handcrafted features, allows building in an unsupervised way, a neural map of an environment from a camera and a compass.^1^

To memorize a place, the LPMP model starts by building a visuospatial code representative of its “spatial configuration,” i.e., all the visual signatures of the landmarks that constitute it and their azimuth angles. It then records it in the connection weights of a neuron called a “spatial” neuron, whose activity indicates the recognition of the memorized place. The LPMP model thus mimics several key structures of the mammalian brain, such as the visual system, and a part of the hippocampal system (Figure 2). This last area is known to be involved in spatial memory processes.

To code a place, the LPMP model starts by extracting *N*_p_ PoIs from the current image *I*, centered on the landmarks that best characterize it (visual system, Figure 2). These PoIs are collected by building a saliency map of *I* *via* the computation of a visual gradient (Deriche filter), followed by a DoG (difference of Gaussian) filter that highlights the curvature points found in the image and ends with a local competition mechanism that selects the most significant PoI. Subsequently, these PoIs are processed one by one by two parallel pathways, the “what” and “where” pathways, which respectively encode for each PoI a visual signature and its spatial orientation (Mishkin et al., 1983). This sequential process mimics the attentional mechanism that allows focusing successively on the more informative regions of the image as observed during the eye saccades in animals (Tsotsos, 1990).

To characterize the visual signature of a PoI, the LPMP model carries out a *log-polar* transform, consisting of the remapping of the pixels around the PoI into log-polar coordinates (log-polar encoding, Figure 2). This remapping allows the system to represent landmarks in a more compact format while producing code that is more robust to small appearance changes induced by the movement of the vehicle (Javier Traver and Bernardino, 2010). The computed signature is then sent to the *winner memory*, WM^l^ (landmark memory, Figure 2), an intermediate memory dedicated to memorizing all the different landmarks encountered.

Composed of *N*_l_ neurons, the WM^l^ memorizes the observed landmarks when the learning of a new place occurs; otherwise, it computes the similarity between the currently observed signature and the already memorized ones. The learning of each landmark is performed by first selecting an available neuron in the layer and by copying the values of the visual signature in the weights of its connection with the log-polar mapping. Thus, the value *N*_l_ defines the maximum number of different landmarks that the memory can encode and is defined at the initialization of the model.

During the computation of the WM^l^ activity, only the *N*_w_ neurons with the highest activity, called the *winning neurons*, remain active and transmit their activity to the next layer of neurons. This filtering process, called “competition,” limits the number of neurons that contribute to the visuospatial code of a place, allowing the system to make multiple hypotheses for a given landmark. Thus, a strict competition allows only a single interpretation of the visual signature, favoring a single hypothesis that could be wrong. Contrarily, relying on a soft competition allows several hypotheses to be taken into account and increases the probability to correctly identify the landmark, but creates noise on the code of a place.

To characterize the spatial orientation of a PoI, the LPMP model computes its “azimuth,” i.e. the absolute orientation of a PoI with respect to the global north. To this end, for each PoI, the “azimuth computation” block shifts the absolute orientation of the vehicle, corresponding to the *x* coordinate of the image center, as a function of the angular deviation of the PoI with respect to this center.

The orientation obtained is then encoded as a *population* of *N*_a_ neurons (Georgopoulos et al., 1988) in the Pl^*a*^ layer (azimuth encoding, Figure 2). More precisely, the activity of each neuron decays exponentially as a function of the angular distance between its preferred direction and the azimuth angle of the currently observed landmark. Consequently, on this Pl^*a*^ layer, a bubble of activity emerges centered at the neuron coding for the azimuth angle of a landmark.

The resulting information of each processing pathway is finally sent to a neural matrix called Max-Pi layer (MPL), which merges the *what* and *where* information of all the landmarks extracted from *I* (what–where merging block, Figure 2). This matrix is uniquely composed of *Max-Pi units*, a specific neuronal structure that, due to its connectivity, performs three operations: a pooling, a product of two modalities, and a temporal integration. Unlike a winner memory (WM), MPL does not learn a pattern and is only used to construct a visual–spatial pattern representative of a place by accumulating the information coming from WM^l^ and Pl^a^.

In the LPMP model, each unit of the same row is connected to a single neuron of WM^l^, encoding a visual signature, and each unit of the same column is connected to *r*_a_ neurons of Pl^a^ [for a final dimension of (*N*_l_ × *N*_a′_)]. Therefore, the learning of a new landmark with WM^l^ is accompanied by the recruitment of a new column of neurons in MPL, so that it can be integrated into the computation of the visuospatial code. The number of columns (*N*_a′_) in MPL defines the number of distinct physical landmarks that could lead to the same visual signature (perceptual aliasing), but nonetheless distinguished by different azimuth angles, that the system can handle.

Therefore, at a given iteration, each Max-Pi unit performs the following three operations:

1. Computing a *max pooling* step on both of its inputs processed *via* a distinct pathway: one is computed on *r*_a_ successive neurons of Pl^a^ and the other one is performed in the WM^l^ layer. However, since each Max-Pi unit is connected to a single neuron of WM^l^, it results in a simple copy of the input activity.
2. Performing a *product* step where both inputs are multiplied.
3. Integrating the value throughout the processing of an image to maintain its activity until the complete processing of the *N*_p_ landmarks selected in *I*.

After having processed all the PoIs of *I*, the final activity of MPL characterizes a spatial configuration of the landmarks specific to a place.

The final activity of MPL is then sent to a second WM called WM^p^ (for winner memory of places), used to memorize the code of the learned images. The activity of this memory allows localizing the system in its environment, behaving in a very similar way to place cells. In the proposed model, the learning of a new image is controlled by a *learning signal*, which synchronizes the learning of a place with the learning of its landmarks.^2^

In its original design, LPMP proposes to autonomously learn an environment *via* a *novelty detector*. This detector automatically triggers the learning of a new place when the activity level of WM^p^ falls below a value *v*, called the *vigilance threshold*. This system leads to an irregular paving of the environment (place fields may have different sizes along a given trajectory), which can be more economical in memory or even more efficient in certain situations (Espada et al., 2019). For the sake of clarity, we call this model the LPMP + Vig model to differentiate it from the version without novelty detector, in which the learning signal is regularly triggered as in conventional VPR systems.

In the following sections, details are given for each of the blocks described above. A table of the parameters and their values is given in the *Appendix* and can serve as a reminder for the notations used.

### 4.2 Visual System

The LPMP visual system is inspired by the mechanisms of visual attention in animals (Treue, 2003; Gaussier and Zrehen, 1995). It performs a non-uniform sampling of the visual input by only extracting visual information on the salient regions *via* a mechanism mimicking ocular saccades (Gaussier and Zrehen, 1995). This method makes it possible to only focus on the most informative parts of an image and could be the solution used by the brain to reduce the computations of its visual system (Tsotsos, 1990).

From a functional point of view, the proposed visual system looks for the most stable high curvature points of an image, e.g., a corner. It begins with a first stage of preparation, where the image *I* is transformed in grayscale and cleaned up *via* a light smoothing and a histogram equalization. Then, a Deriche filter (Deriche, 1987) is used to highlight the edges of the image. This filter is a variant of the Canny filter (Canny, 1986), which incorporates a smoothing, and whose impulse responses are given by:

$$f\left(x\right)=kxe^{-\alpha |x|}$$ (1)

With *α* as the smoothness parameter between 0 and 1 and *k* is a constant. Decreasing *α* increases smoothing and improves the edge detection to the detriment of their localization and *vice versa*.

Subsequently, a convolution with a DoG is used on the image to highlight the curvature points of the different edges detected. This second filter is constructed by subtracting two Gaussians of different widths (standard deviations), as described in the following equation:

$$\Gamma {\left(x,y\right)}_{\sigma_{1},\sigma_{2}}≜\frac{1}{\sqrt{2\pi }}\left(\frac{1}{\sigma_{1}}e^{-\left(x^{2}+y^{2}\right)/2\sigma_{1}^{2}}-\frac{1}{\sigma_{2}}e^{-\left(x^{2}+y^{2}\right)/2\sigma_{2}^{2}}\right)$$ (2)

with *σ*_1_ and *σ*_2_ as the standard deviations of the two Gaussians. The use of a DoG filter on the image allows diffusing its edges, reinforcing their value at the curvature positions, as described in Gaussier and Cocquerez (1992). The succession of these two filters builds a saliency map *S*, which highlights the important curvature points of *I*.

Finally, a *local competition* is carried out between the local maxima of *S* to extract *N*_p_ stable PoIs from *I* (see Algorithm 1). This competition consists of successively selecting the most active points in *S* while inhibiting the other points around, in a radius defined by *r*_c_. This exclusion radius prevents the system from selecting values on the saliency map around positions already selected. The last step consists of only keeping the *N*_p_ PoIs with maximal saliency values.

Algorithm 1 Competition on saliency map.

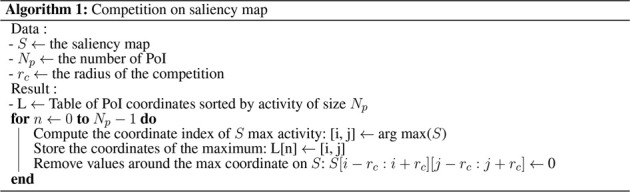

### 4.3 Log-Polar Encoding

The log-polar encoding module was used to characterize the visual information of a PoI by mimicking the functioning of the eyes (Schwartz, 1980; Araujo and Dias, 1997). Indeed, this encoding technique is inspired by “cortical magnification,” i.e., the very specific structure of the retina (particularly the fovea) where the visual receptive field spacing and size increase with the distance from the central part of the retina.

The log-polar mapping starts with the extraction of a circular image patch (or vignette) of radius *r*_max_ around a specific PoI, then is followed by the computation of a non-constant sampling where the number of sampled pixels increases with the distance to the center of the vignette (see Figure 4).

**FIGURE 3:**
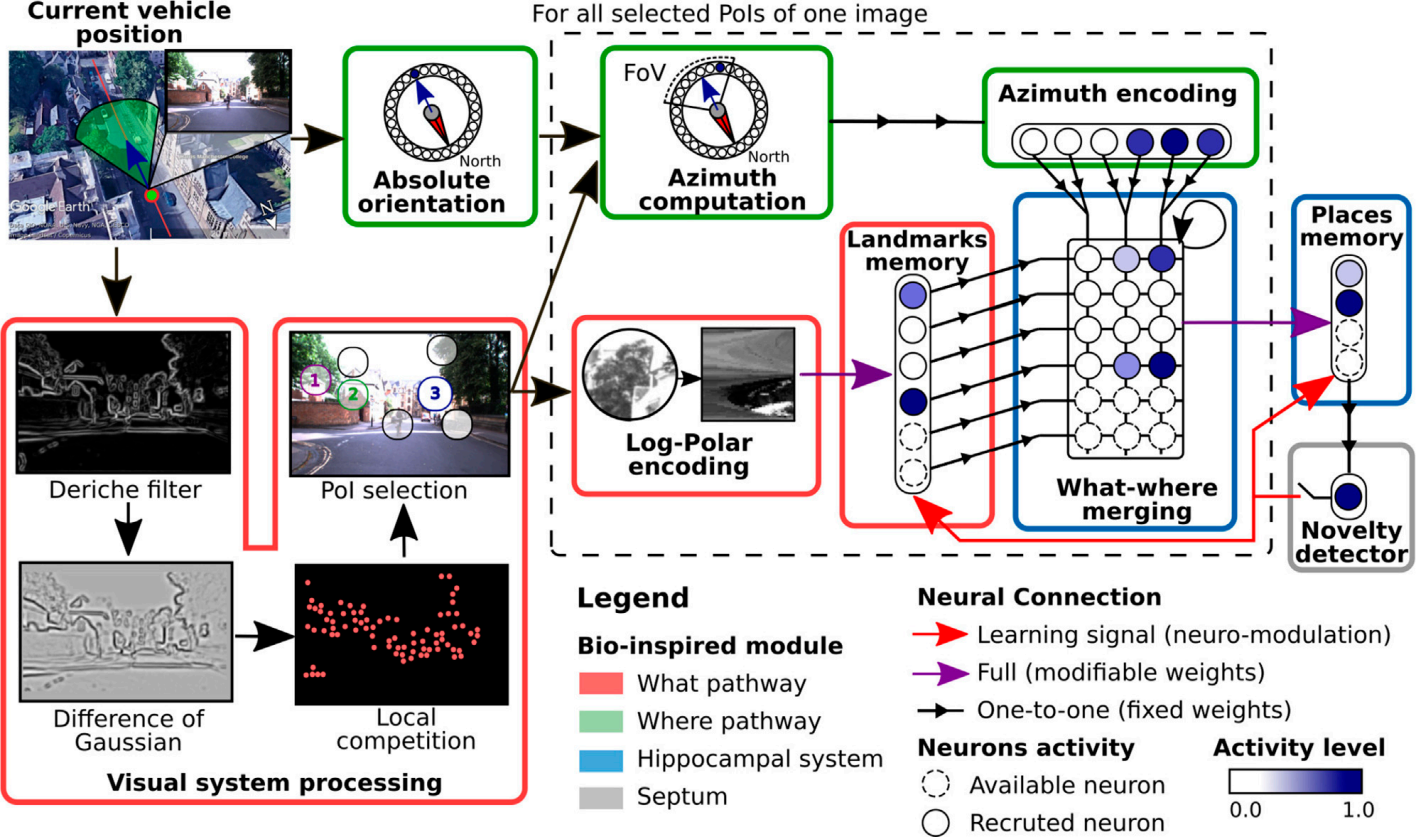
Complete scheme of the Log-Polar Max-Pi (LPMP) model. This figure illustrates how the LPMP model builds a place representation from one image of a route sequence. In this neural model, a place is defined as a visuospatial pattern specific of the current location and results from the merging of two pieces of information: the image *I* and the compass of the vehicle. To characterize a visual scene, LPMP detects *N*_p_ points of interest (PoIs) from an image. Subsequently, in an attentional loop, the system sequentially extracts image patches centered on each PoI, computes a feature vector (*via* a log-polar transform), and learns this pattern in a first neural winner memory (landmark memory). In parallel, their spatial orientation (according to a given absolute reference) is computed in the azimuth computation block and mapped in a neural layer (azimuth encoding). Then, both pieces of information are then merged in a neural Max-Pi layer (what–where merging). When all the selected PoIs are processed, the resulting activity pattern of the Max-Pi layer is representative of the spatial configuration of the landmarks and is therefore characteristic of the corresponding place. Finally, this pattern is stored in a second neural memory (winner memory), called place memory. A vigilance system makes it possible to automatically build a map of the environment by triggering the recruitment and the learning of new neurons to code the new locations visited. This decision is based on the level of activity of the winner memory, which codes for the already known places.

**FIGURE 4:**
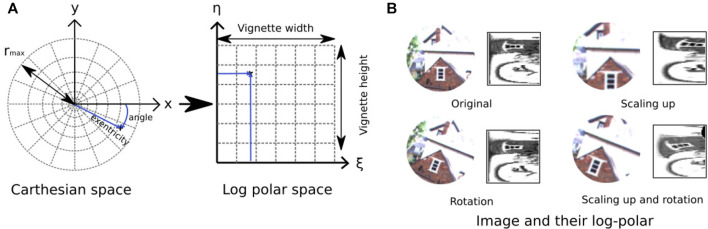
Illustration of log-polar transform. **(A)** Log-polar transformation converts a circular vignette of size *r*_max_ from the 2D Cartesian coordinate system into the log-polar coordinate system with *ξ* (eccentricity) and *η* (angle). **(B)** Examples of log-polar code for three transformations (rotation, scaling up, and rotation combined with scaling up). *Left*, input image; *right*, log-polar code.

From a mathematical point of view, the log polar transformation corresponds to the remapping of the vignette from the 2D Cartesian coordinate system (*x*, *y*) to the log-polar coordinate system. It can be expressed by the following equation:

$$\xi =\log\sqrt{x^{2}+y^{2}},\;\eta =\mathrm{arctangent}\left(y,x\right)$$ (3)

where *ξ* (the eccentricity or magnitude) and *η* (the angle) are the coordinates in the log-polar space.

With the visual space representation not being continuous, the circular vignette is discretized into a “visual sector,” according to the dimension of the output matrix (*D*_e_ × *D*_a_). The pixels of a given visual sector are represented in a cell of the output matrix, usually *via* an average weight function.

This transformation allows coding the vignette in a more compact format than with a constant resolution for a very low computational cost (Javier Traver and Bernardino, 2010). In mobile robotics, relying on log-polar transformation provides robustness to small appearance changes of landmarks induced by a small variation of point of view when the robot moves, as shown in previous indoor and off-road experiences (Joulain et al., 1997; Giovannangeli et al., 2006b; Belkaid et al., 2016).

### 4.4 Azimuth Computation and Encoding

The processing of the *where* information (or spatial signature) is performed by the *azimuth computation* and *azimuth encoding* modules. It corresponds to the absolute orientation of the landmark, according to a fixed reference (e.g., the magnetic north). This information can be found in the brain under the form of head direction cells, neurons that activate when the animal directs its head in a specific direction. Note that an absolute reference is needed to build an allothetic representation of place independently of the current orientation of the vehicle, e.g., the north direction retrieved with a magnetic compass. For the sake of simplicity, the orientation information in this work is derived from the vehicle log of the datasets. But in order to only deal with the visual modality, the orientation information could also be derived from the visual input *via* a visual compass (Gourichon et al., 2003; Delarboulas et al., 2014).

To compute the azimuth angle $\theta_{l}^{north}$ of a landmark, the system shifts the absolute angle of the vehicle $\theta_{Vehicle}^{north}$ with respect to the magnetic north^3^ by the angle of the POI calculated in the egocentric frame of reference, i.e., relative to the center of the image. $\theta_{poi}^{ego}$ is computed in function of *x* the horizontal position of the landmark in image *I* of dimension (*D*_w_ × *D*_h_) and the width of the field of view (FOV) *θ*_fov_.

$$\begin{array}{c} \theta_{l}^{north}=\theta_{poi}^{ego}+\theta_{Vehicle}^{north}\;\left(\mathrm{mod}\;2\pi \right)\;\text{with}\;\theta_{poi}^{ego}=\theta_{fov}\left(\frac{x}{D_{w}}-0.5\right) \end{array}$$ (4)

Then, this information is encoded in the Pl^a^ layer by a population of neurons (Georgopoulos et al., 1988), a neural structure within which a bubble of activity emerges centered at the neuron coding for the azimuth angle of a landmark. More precisely, each of its *N*_a_ neurons exhibits a maximal response to a preferred azimuth angle. Their activity then decays exponentially as a function of the angular distance between their preferred direction and the azimuth angle of the currently observed landmark:

$$Pl_{j}^{a}=\exp-\frac{{\left(\left(\left(j-\theta \right)\mathrm{mod}N_{a}\right)-N_{a}/2\right)}^{2}}{2\sigma_{azim}^{2}}$$ (5)

where $Pl_{j}^{a}$ is the activity of the *j*th neuron on azimuth layer **a** and *σ*_azim_ is the standard deviation of the Gaussian.

### 4.5 Winner Memory Layer

The LPMP model requires the use of two memories to store the log-polar code of landmarks (landmark memory, Figure 3) and the activity of the Max-Pi layer (place memory, Figure 3).

In its current formulation, the LPMP model uses a WM (Espada et al., 2019), a simple neural model of memory that allows storing vectors of data in one iteration. The use of one-shot learning is one of the keys to the effectiveness of the model, a peculiarity considered as one of the key properties of the memory of animals (Lee et al., 2015). From a functional point of view, the network stores directly in the weights of these neurons a set of data, with one neuron per different signal, which leads to a strong reduction at the output.

This memory has thus two advantages:

• It learns in a single iteration a vector (for example, the log-polar code of a landmark), allowing for fast learning without the need of pre-training (one-shot learning principle).
• It allows applying a form of filtering on the reading of the memory by allowing only the strongest neurons to express themselves (competition principle).

From a structural point of view, a WM network is composed of *N*_total_ neurons with *u* learned neurons and *N*_total_ − *u* available neurons (i.e., with null weights^4^). To record a new entry, a learning signal *λ*_*i*_(*t*) is sent to the network, which saves the information directly in the weights of the next available neuron *u*. Thus, the updated formula of a neuron *i* ∈ [0, *N*_total_ − 1] when learning a pattern **d** of size *N*_d_ can be written as:

$$w_{i,j}\left(t\right)=\left\{\begin{array}{cc} d_{j}\left(t\right),\; & \text{if }\lambda_{i}\left(t\right)=1,\\ w_{i,j}\left(t-1\right),\; & \text{otherwise}. \end{array}\right.$$ (6)

with *w*_*i*,*j*_ the weight matrix of the WM and *d*_*j*_ the *j*th element of **d**.

Two steps are necessary to compute the final WM activity: a comparison step, where the activity of each neuron represents the degree of similarity (distance) of its learned pattern with the input **d**, and a competition step, where only the most active neurons remain active. The computation of the activity of a neuron, *i*, before competition is written as:

$$\begin{array}{c} {\hat{s}}_{i}\left(t\right)=1-\frac{1}{K_{1}}\overset{N_{d}}{\underset{j=0}{\sum }}h\left(w_{ij},d_{j}\left(t\right)\right)\left(w_{i,j}>k_{1}\right)\;\text{ with }K_{1}=\overset{N_{d}}{\underset{j=0}{\sum }}\left|w_{i,j}>k_{1}\right| \end{array}$$ (7)

with *h*(*w*_*ij*_, *d*_*j*_(*t*)) = ‖*w*_*ij*_ − *d*_*j*_(*t*)‖ a Euclidean distance between the data **d** and the weight of the *i*th neuron. *K*_1_ (Eq. 7) is a normalization factor based on the number of activated weights, i.e., weights greater than an activity threshold expressed by the constant *k*_1_. In this equation, weights less than *k*_1_ are excluded to skip the distance computation between the input and their weight, which would lead to an irrelevant (small) activity. A neuron *i* has, therefore, maximum activity when the pattern is close to its weight. An implementation of the $\hat{s}\left(t\right)$ computation on the WM is proposed in Algorithm 2.

Moreover, there are several ways to perform a competition on the WM. In our case, we chose to use a basic competition mechanism where only the *N*_w_ neurons with the highest activities are expressed in the final activity $s_{i}\left(t\right)=c\left(\hat{s_{i}}\left(t\right),N_{w}\right)$ with *c* as the competition function.

Algorithm 2 Computation of $\hat{s}\left(t\right)$ on WM.

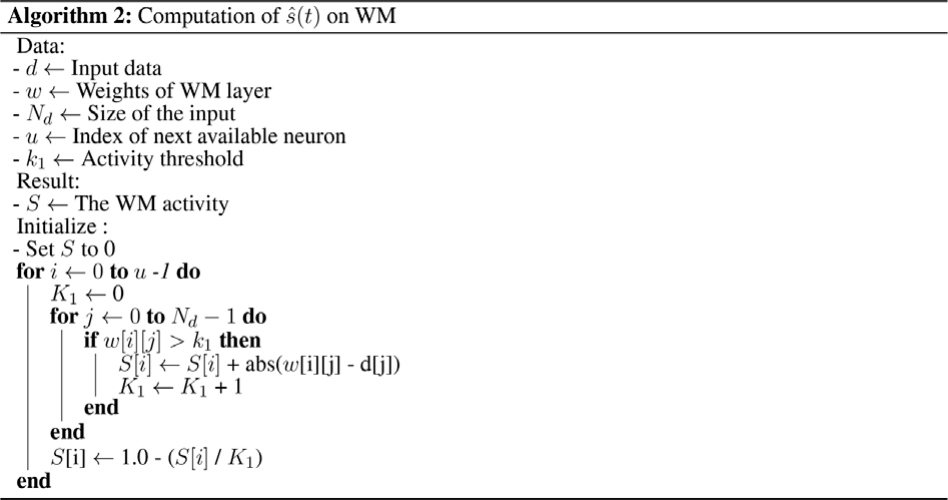

### 4.6 Max-Pi Layer

**FIGURE 5:**
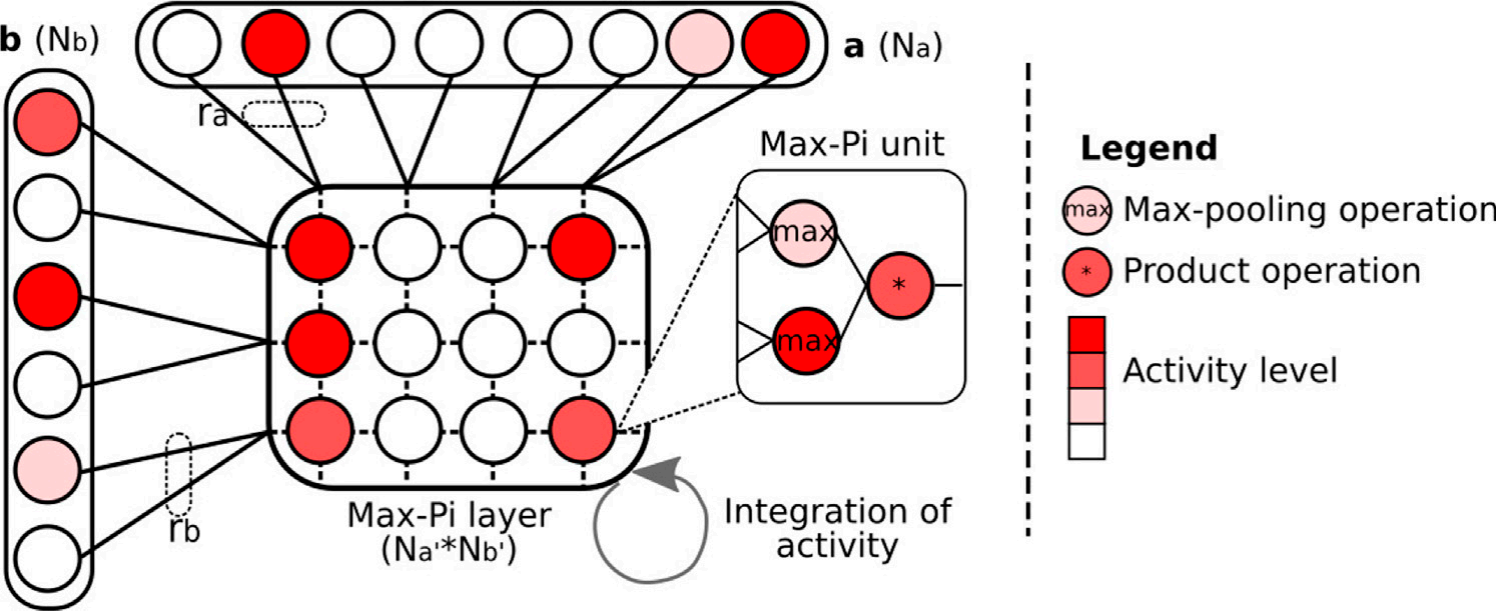
Illustration of the Max-Pi layer (MPL) operation. To merge information coming from two vectors **a** and **b**, the MPL performs three operations: a max pooling on *r*_a_ and *r*_b_ neurons, a merging on the results of the max pooling *via* a product, and a temporal integration over a given number of iterations. These operations allow the information coming from vectors **a** and **b** to be combined into a compressed activity pattern.

The Max-Pi layer (MPL) is a neuronal structure used in the LPMP model to merge and integrate the information coming from the *azimuth encoding* block and the *landmarks memory* block for the duration of an image (see Figure 3). This neuron layer, taking the form of a 2D matrix, is composed of Max-Pi units, specific neuronal units that, because of their connections, carry out three processing operations: a max pooling, a merging *via* a product, and a temporal integration (see Figure 5).

To describe the operations realized by MPL, let us consider a matrix *x* composed of *N*_a′_ × *N*_b′_ Max-Pi units, and two neuron vectors, **a** of size *N*_a_ and **b** of size *N*_b_. Then, the activity of neuron *x*_*i*,*j*_ (the *i*th row and *j*th column) resulting from vectors **a** and **b** at time *t* can be expressed as:

$$x_{i,j}\left(t\right)=x_{i,j}\left(t-1\right)\left(1-R\left(t\right)\right)+\underset{k=\left[r_{a}.i,r_{a}.\left(i+1\right)\right]}{\max}\left(a_{k}\left(t\right)\right)\underset{l=\left[r_{b}.j,r_{b}.\left(j+1\right)\right]}{\max}\left(b_{l}\left(t\right)\right)$$ (8)

In this equation, the first term allows accumulating the activity of *x*_*i*,*j*_ over time. The function *R*(*t*) is a binary signal that allows erasing the content of the matrix (typically after processing all the PoIs in an image). The second term expresses the max fusion between **a** and **b**. Thus, each neuron in this matrix merges *r*_a_ and *r*_b_ neurons, expressible by $r_{a}=\frac{N_{a}}{N_{a^{\prime }}}$ for **a** and $r_{b}=\frac{N_{b}}{N_{b^{\prime }}}$ for **b**.

The MPL is inspired by the functioning of the cortical columns and, more specifically, by the Sigma–Pi units (Mel and Koch, 1990; Plate, 2000), neuronal structures performing a processing close to the one of Max-Pi units. The essential difference between these two structures is that a Sigma–Pi unit realizes the addition of a multiplicative cluster of neurons (called pi-neurons), where the Max-Pi unit will rather use a pooling formula followed by a multiplication, which is easier to manage from a computational point of view.

In the LPMP model, the dimension of the vector **b**, i.e., the WM^l^ layer, changes during the execution of the model. Indeed, the number of landmarks evolves as the LPMP model memorizes new locations. Thus, when a new landmark is added in WM^l^, a new column of neurons is recruited in MPL. By this way, the new landmark is integrated into the activity of the MPL matrix, which becomes able to encode richer places.

The MPL was implemented using an intermediate max-pooling layer to save computational costs, as described in Algorithm 3.

Algorithm 3 Computation of Max-Pi activity.

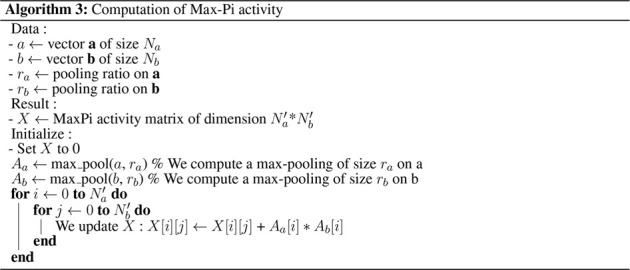

### 4.7 Novelty Detector and LPMP + Vig

The *novelty detector* allows automatically controlling the learning signal of the model in order to generate a representation of an environment in an unsupervised way. This mechanism is inspired by the ART (adaptive resonance theory) model (Carpenter et al., 1991), an important model of neuroscience that explains how the brain can autonomously learn to categorize information in a dynamic world.

To trigger the learning signal, the novelty detector simply checks whether the recognition level of the current place, represented by the highest value of WM^p^, falls below a constant, called the *vigilance threshold*. Thanks to this system, the LPMP model generates an irregular representation of an environment, which adapts to its complexity (Espada et al., 2019). Thus, such a system can reduce the representation cost of an environment^5^ or improve the localization performance, especially in complex ones that require a strong sampling of the sequence.

To keep the consistency of the learned representation, the model relies on the same and single signal to trigger both the learning of a new place in WM^p^ and the learning of corresponding landmarks in WM^l^.

## 5 Experiments

### 5.1 Datasets

To evaluate the performance of the LPMP model, we decided to use the Oxford car dataset (Maddern et al., 2017; Maddern et al., 2020), a recent self-driving car dataset intended for VPR models. This dataset is composed of a hundred of records made over 9 km in the city of Oxford and provides access to trajectories under a great variety of conditions such as season, weather, and traffic. Due to its very large size, the dataset is often cut to be used in the evaluation of VPR systems (Garg et al., 2018; Sattler et al., 2018; Chancán and Milford, 2020; Pan et al., 2020). Although several papers have presented results on this dataset, a few of them indicated which subset of the dataset was used,^6^ requiring us to propose our own test benchmark.

**FIGURE 6:**
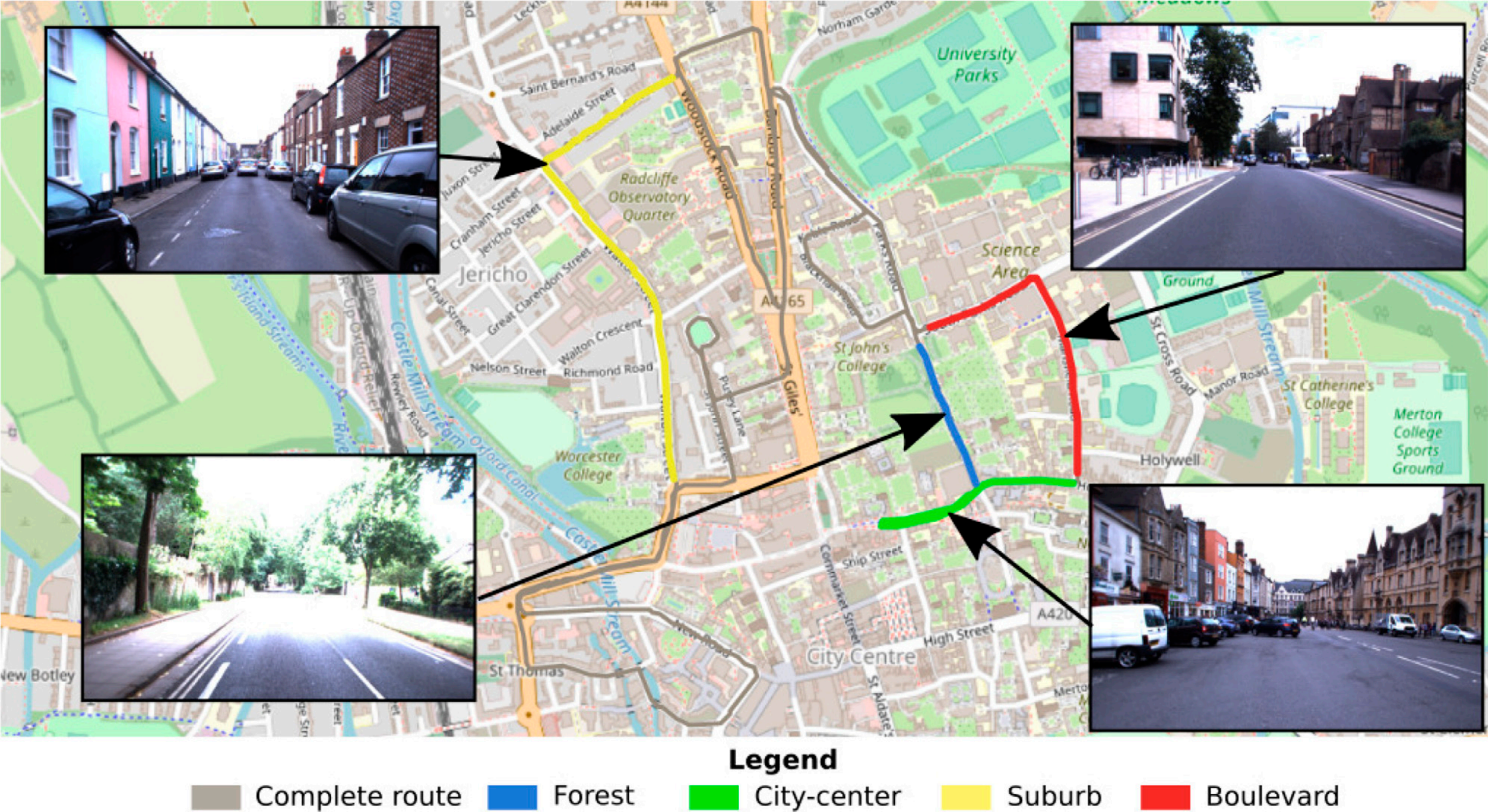
Illustration of the test benchmark dataset. The proposed test benchmark is composed of four kinds of routes covering the following urban environments: *suburb*, *forest*, *boulevard*, and *city center*. These routes were selected from the entire *Oxford car dataset* to perform a comparison of the performance of visual place recognition (VPR) models according to the environment.

In this paper, we decided to compare the performance of the models according to the type of environment. Four “routes” (each composed of four trajectories/recordings; see below) were extracted from the whole dataset, which go through four different types of environments: *city center*, *suburb*, *boulevard*, and *forest* (see Figure 6). Particular attention has been paid to take the longest possible sequences presenting a well-defined environment, as illustrated in the figure (see Figure 6).

For each route, four recordings at different times were selected and therefore presented. To the extent of what is available, the recordings include different levels of human activities that allow testing the robustness of the different algorithms. To measure this activity, we used Yolo, an image classification algorithm (Redmon et al., 2016), to automatically count the number of vehicles and pedestrians.

The different recordings were selected in order to have comparable recordings, i.e., with identical trajectories and without sensor problems (many sequences had to be eliminated because of GPS problems). Moreover, the sequences were chosen under equal weather and seasonal conditions in order to focus our analysis only on the localization performance achieved in the different environments. Table 1 presents the retained sequence division. The datasets used for our evaluation represent around 10 km of trajectories from the original Oxford car dataset.

**TABLE 1:** Different trajectories selected from the *Oxford car dataset*

Environment	Images	Distance (m)	Duration (s)	Activity rate	Sequence date	Index on reference sequence
Boulevard	1,–930	632	125	4.5	2014/07/14 15:42:55	2,552–4,481
Boulevard	1,401	625	89	6.8	2014/07/14 14:49:50	2,820–4,220
Boulevard	1,159	624	74	7.0	2015/07/29 13:09:26	5,928–7,086
Boulevard	1,572	626	101	7.1	2015/08/4 14:54:57	5,665–7,236
City center	1,521	532	104	10.6	2015/05/19 14:06:38	6,199–7,719
City center	1904	569	124	10.9	2015/08/14 14:54:57	7,431–9,334
City center	2,227	527	143	12.0	2015/07/29 13:09:26	7,210–9,436
City center	2,134	585	140	13.5	2015/05/22 11:14:30	7,728–9,861
Forest	927	292	61	5.9	2014/07/14 14:49:50	5,190–6,116
Forest	828	289	54	6.2	2015/08/14 14:54:57	9,563–10 ,390
Forest	566	286	38	6.6	2015/05/19 14:06:38	7,827–8,392
Forest	595	287	37	8.6	2015/05/22 11:14:30	10 ,211–10 ,805
Suburb	3,606	1,011	248	6.2	2015/04/24 08:15:07	17 ,342–20 ,947
Suburb	3,472	1,029	233	6.4	2014/07/14 15:42:55	8,965–12 ,436
Suburb	4,485	1,013	329	6.8	2015/05/19 14:06:38	13 ,100–17 ,584
Suburb	3,655	1,014	239	7.2	2015/05/22 11:14:30	15 ,164–18 ,815

The table presents the trajectories selected by environment in Oxford and their characteristics. The urban activity metric corresponds to the average of “urban elements” (i.e., car, pedestrian) per image detected by the Yolo network (Redmon et al., 2016).

### 5.2 Evaluation Methodology

The experiments carried out in this paper followed a standard procedure of place recognition evaluation (Sattler et al., 2018) under two criteria: localization performance and computational performance.

#### 5.2.1 Localization Performance

To obtain reliable results, we decided to perform our experiments by environment in cross-validation. Thus, for one experiment, two trajectories of the same environment are selected and are treated in four stages:

• Learning an environment: The images of one trajectory (the *learning trajectory*) are subsampled every *d*_samp_ meter and used by the model to build its representation of the space.
• Query set: A set of *N*_queries_ images is randomly selected on the second trajectory (the *test trajectory*) and is presented to the model. In this paper, we chose to use an *N*_queries_ that represents 25% of the test trajectory.^7^
• Hypothesis construction: For each query, the system returns the index of the images considered closest to the request (the hypotheses).
• Query evaluation: The quality of the answer of the VPR model is assessed by computing whether the distance between the GPS coordinates of a query and the GPS coordinates of the best hypotheses is less than a threshold, *d*_err_.

We have thus chosen to measure the performance of the model using three sampling distances of 2, 5, and 10 m. Thus, for each environment, 36 experiments were conducted to cover the different sampling distances and all the possible learning and test trajectory configurations.

We used an error threshold equal to half the sampling distance (perfect location) with a tolerance of 15%, such as a *d*_err_ = *d*_samp_ × 0.65. This method establishes the case of a positive location to a space of size *d*_samp_, centered on the coordinate of the places with a tolerance of 15% on each side.

#### 5.2.2 Computational Cost

The computational cost was evaluated by measuring the computational time that each model takes on average to answer a query as a function of the number of locations learned. The models were evaluated with a controlled number of CPUs and no GPU to limit as much as possible (even though not entirely) the impact of code optimization on each model.

#### 5.2.3 Comparison With the State of the Art

The LPMP model has been compared to two major models of the state of the art: CoHog, a handcrafted feature model (Zaffar et al., 2020b), and NetVLAD, a CNN model (Arandjelović et al., 2016). The two models are both, in their respective fields, the best-performing ones on localization tasks (see review in Zaffar et al., 2020a).

It should be noted that the LPMP model, unlike the NetVlad and CoHog models, uses the absolute orientation of the vehicle to encode a place. For the sake of simplicity, this information is directly obtained *via* a magnetic compass integrated on the self-driving vehicle. However, it would have been possible to use a visual compass, as in a previous work (Delarboulas et al., 2014).

#### 5.2.4 Evaluation of LPMP + Vig

**FIGURE 7:**
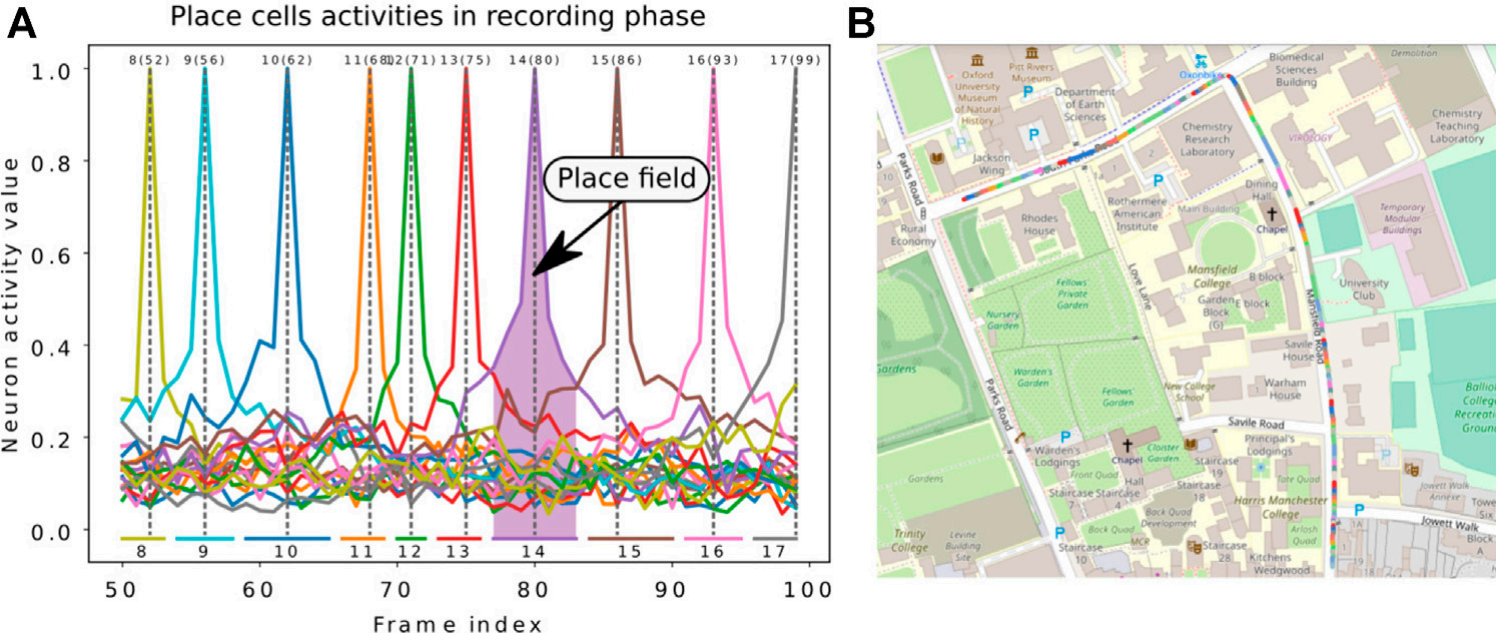
Illustration of the Log-Polar Max-Pi (LPMP) + Vig problem. **(A)** The *left image* presents the neural activity of the winner memory layer, generated during the recording step. During this phase, no learning occurred in order to record which cell should respond to a given position. Thus, the *gray dotted lines* indicate the frame index at which learning occurred for each neuron, the *numbers on top* denote the index of neurons, and the *numbers in brackets* indicate the index of the corresponding image in the sequence. The *colored horizontal bars* indicate the spaces encoded by each neuron. **(B)** The second image shows a map representing one recording of the *Oxford car dataset* with the learned places. *Each colored line* represents the space computed *via* the replay-learn evaluation (RLE) method, according to **(A)**.

For the LPMP + Vig version, the proposed test pipeline cannot be directly used due to the vigilance system. Spatial neurons are automatically generated by traversing a sequence and therefore construct a neural map of an environment, i.e., made up of place cells with variable place field sizes (see Figure 7) since the learning of a new place depends only on how much the visual appearance of the scene varies. This non-regularity of the paving makes it difficult to produce a realistic assessment of the model that does not penalize or over-values the obtained scores.

To solve this problem, we proposed the method RLE (for Replay-Learn Evaluation) to estimate the size of the spaces encoded by the model. The idea of the method was to replay the learning sequence on the model after learning (in a phase called evaluation) to look, image by image, at which neurons were activated.

Thus, the RLE method is divided into three phases:

• Exploratory step: Neurons are learned sequentially while processing the trajectory number 1, according to the recognition threshold set. Due to the causal nature of the learning process, only half of each place field can be computed during this stage (the answer of the neuron once learned). But once learned, each neuron can also respond on the frames preceding the one used for its learning (first half of the place field).
• Recording step: Once the trajectory number 1 is finished, it is replayed so that the full place field can be computed for each neuron that learned a place.
• Measure step: Just as with the previously described methodology, the second trajectory is processed to measure the localization performance while performing place recognition. This second trajectory is on the same road as trajectory 1, but performed at a different moment and not at exactly the same GPS coordinates.

Thus, this method allows associating a neuron with portions of the trajectory (Figure 7). These spaces can be used to precisely determine the quality of the localization by comparing them with the activity of the neurons during the recording step.

Moreover, to obtain a comparison with algorithms without a vigilance loop, we aligned the results according to the place sampling rate and the average size of the generated place fields. This method requires exploring several values of vigilance to find a value that approaches the desired sampling rate value.

### 5.3 Metrics

To assess the performance of the different models, we used standard precision/recall measurements, summarized by the areas under the curve (AUCs) and the recall at 100% precision (Sattler et al., 2018). The large amount of tests carried out in this paper (576 experiments) forced us to present average displays, reflecting the average performances and not the best possible values.

### 5.4 Implementation Details

To make the comparison with CoHog and NetVLAD, we used the original implementation and parameters of the authors in python. For the NetVLAD network, we used the best pre-trained model proposed by the author (VGG-16 + NetVLAD + whitening), trained on the Pittsburgh dataset and considered to give very competitive results on VPR issues (Arandjelović et al., 2018) [see Zaffar et al. (2020a) and Zhang et al. (2021) for more details].

The parameters used in the LPMP are those of the reference implementation of LPMP (Espada et al., 2019) and are given in Table 2. A region of interest (ROI) has been added to all the systems tested (LPMP, CoHog, and NetVLAD) to remove part of the sidewalk, carrying little information. This treatment has been done to the advantage of all models for the sake of equality.

**TABLE 2:** Log-Polar Max-Pi (LPMP) model parameters

**Parameter name**	**Part**	**Description**	**Value**
(*D*_w_ × *D*_h_)	Acquisition	Image dimension (pixels)	640*400
*N*_p_	Visual system	Number of PoIs	50
ROI	Visual system	Region of interest applied when selecting PoIs (pixels) organized as follow (*x*_1_, *y*_1_, *x*_2_, *y*_2_)	(0, 0, 640, 250)
*α*	Visual system	Deriche filter (pixels)	0.4
*σ*_1_	Visual system	Dog filter (pixels)	2
*σ*_2_	Visual system	Dog filter (pixels)	8
*r*_c_	Visual system	Competition radius (pixels)	16
*σ*_azim_	Azimuth computation	Azimuth diffusion	0.5
*N*_a_	Azimuth encoding	Number of azimuth angles	360
*r*_max_	Log-polar encoding	Max log-polar radius (pixels)	60
(*D*_e_ × *D*_a_)	Log-polar encoding	Log-polar signature dimension (pixels)	50 × 50
*k*_1_	Winner memory	Activity threshold	0.1
*N*_l_	Landmark memory	Number of winners	50
*N*_a′_	Max-Pi layer	MPL column number (neurons)	3
*r*_a_	Max-Pi layer	Pooling ratio (neurons)	120
*N*_p_	Place memory	Number of winners	1

Main parameters used in the LPMP model. Their values come from previous work on the LPMP model (Espada et al., 2019). *PoIs*, points of interest

Performance experiments were carried out using an AMD Ryzen Threadripper 2990wx (3.7 GHz). The experiments on computational cost were carried out using an Intel Core i9-9880H (2.3 Ghz).

## 6 Results

### 6.1 Evaluation of Localization Performance With LPMP

**FIGURE 8:**
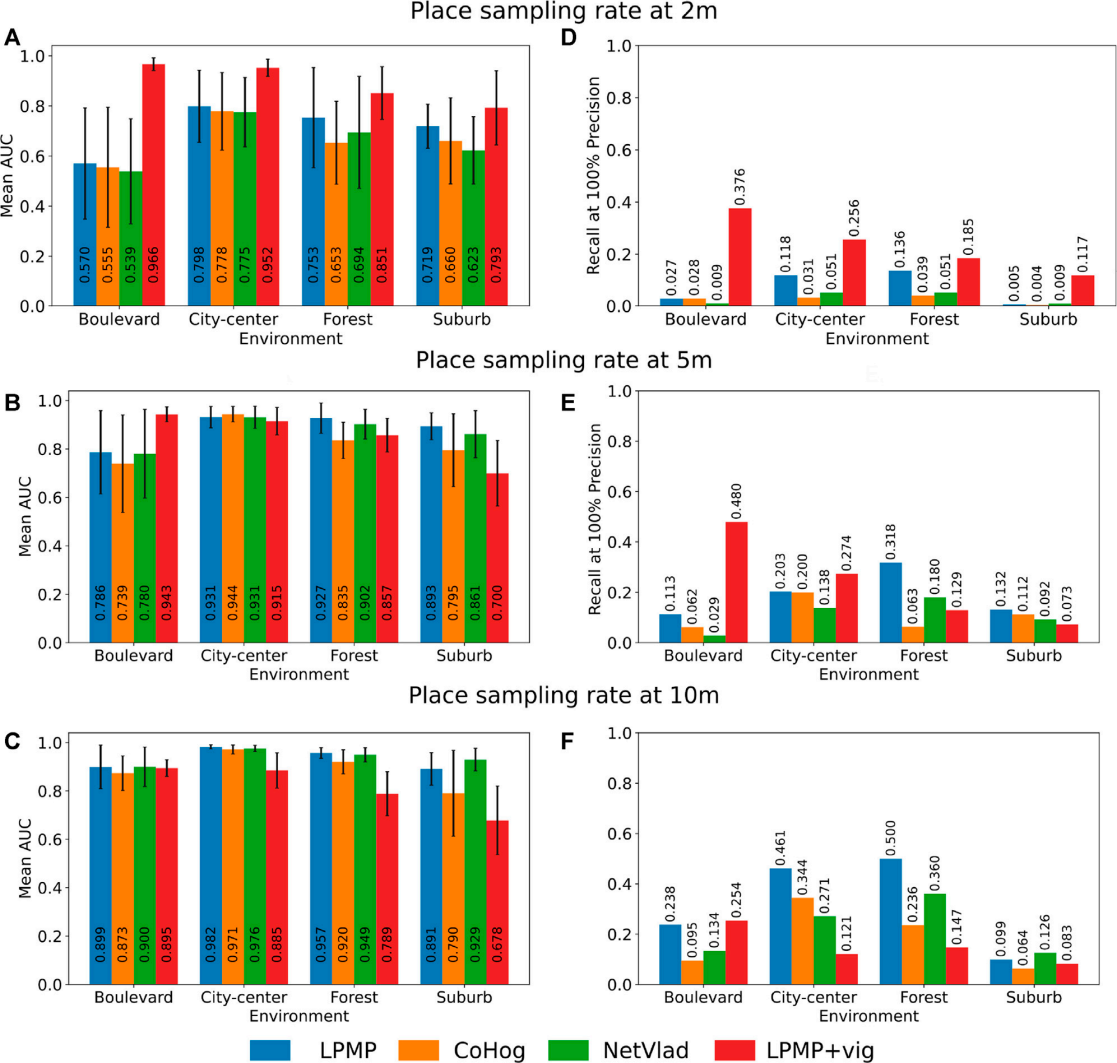
Localization performance of the Log-Polar Max-Pi (LPMP), LPMP + Vig, CoHog, and NetVLAD models. Each model is evaluated in cross-validation on each environment with three place sampling rates. Localization performance was assessed by computing standard precision/recall curves, summarized by the areas under the curve **(A–C)** and their recall at 100% precision **(D–F)**. For the LPMP + Vig model, which autonomously learns when to learn an image on a sequence, the score was computed using the replay-learn evaluation (RLE) method, a method for estimating the spaces encoded by the model that takes into account the non-regularity of the representation. The standard deviation is indicated by a *horizontal black bar*.

Figure 8 shows the average performance of LPMP, LPMP + Vig, CoHog, and NetVLAD according to the place sampling rates and the environments. Thus, the graphs in Figures 8A–C present the mean AUC of the precision–recall curves according to each environment and place sampling rate. The graphs in Figures 8D–F show the recall at 100% precision of the precision–recall curves and serve as a complement to the AUC measurement. Due to the application of a cross-validation method, each value presented is therefore the average of 12 values computed on the precision–recall curves to cover every possible combination of learning/test sequences by environment and by sampling rate.

As shown in Figures 8A–C, the LPMP model gave, in most tests, higher mean AUC values than those of CoHog and NetVLAD: on average 6% more efficient than CoHog and 4% better than NetVLAD. One can notice two cases in which the LPMP model did not provide the best results: the *city center* environment with a place sampling rate of 5 m (see graph in Figure 8B) and the *suburb* environment with a place sampling rate of 10 m (see graph in Figure 8C).

Secondly, the graphs in Figures 8D–F present similar results with the mean recall at 100%: on average, LPMP had a recall at 100% on average 10% better than CoHog and 13% better than NetVLAD. The model did not give the best results only on the *suburb* environment (graph in Figure 8F).

Thirdly, one can notice that LPMP, CoHog, and NetVLAD followed a common dynamics in terms of localization performance by environment: on all learning sampling sizes, these three models gave the best performance on *city center*, followed by *forest* and *suburb* and *boulevard*, which seem to be particularly difficult for VPR models with a fixed sampling rate. For example, the graph in Figure 8B shows that LPMP, CoHog, and NetVLAD together gave average AUCs of 0.935 on *city center*, 0.897 on *forest*, 0.768 on *boulevard*, and 0.849 on *suburb*, confirming this trend.

This difference can be explained by the greater wealth of visual information in the *suburb*, *city center*, and *forest* environments than that in the *boulevard* environment, which is more monotonous and therefore more difficult to precisely characterize. The graphs in Figures 8D–F confirm this trend, at the difference that the best recalls were obtained on *forest* and not *city center*.

### 6.2 Evaluation of Localization Performance With LPMP + Vig

As shown in the Figures 8A–C, the LPMP + Vig model gave better performance than did LPMP, CoHog, and NetVLAD on a high sampling rate. Thus, LPMP + Vig improved the performance of LPMP on average by 20% in the graph in Figure 8A (with a maximum improvement of 70% in performance on *boulevard*), suggesting that VPR models can benefit from the use of a vigilance system when considering high sampling rates.

Indeed, the use of a vigilance system allows creating places according to the richness of the environment, which limits the learning of a similar place by producing a dynamic paving and consequently limits the redundancy in the environment representation. This gain in performance, however, disappears with a low place sampling, as visible in the graph in Figure 8B, C. In this situation, the vigilance system creates neurons that code larger spaces, causing loss of precision that degrades the performance.

Secondly, one can notice that the LPMP + Vig model had a different dynamic by environment compared to models based on regular sampling, showing its best results on *boulevard*, then on *city center*, *forest*, and *suburb*. Indeed, the LPMP + Vig model remained efficient on all sampling with the *boulevard* environment, contrary to the *suburb* environment where the localization performance decreased rather quickly.

This phenomenon can be explained by the proportion of the distal and proximal landmarks in the field of vision, according to the different environments. When the vehicle moves, the appearance changes of the landmarks depend on their distance to the vehicle. Indeed, the visual appearance of distal landmarks varies less than that for proximal ones.

Moreover, the presence of distal landmarks is related to the openness of the field of vision offered by the different environments. Indeed, the *boulevard* environment is characterized by a more open FOV than the *suburb* environment. Consequently, since a sufficient number of distal landmarks can be found in the *boulevard* sequence, the LPMP + Vig model can exhibit a stable level of place recognition, leading to a paving of the environment that allows for better localization performance.

On the contrary, in the *narrow* *suburb* environment, the FOV is restricted by the presence of closer elements (buildings, vehicles, etc.). The presence of these numerous proximal landmarks causes strong variations in the level of recognition, making the automatic paving of the environment more difficult to achieve. This result is consistent with previous studies of this model on the KITTI dataset (Espada et al., 2019).

### 6.3 Dynamics of the Vigilance System

**FIGURE 9:**
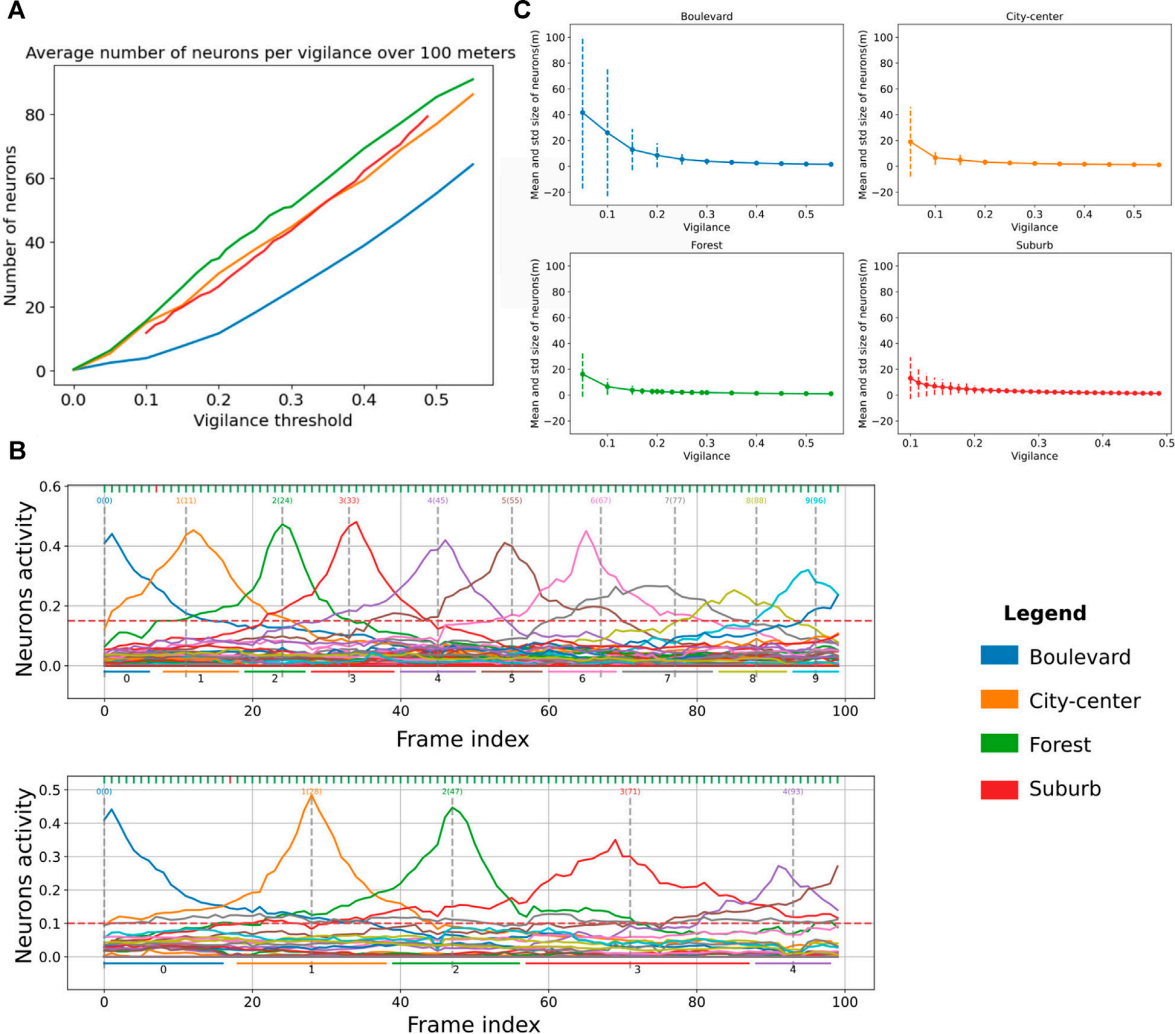
Evaluation graphs of Log-Polar Max-Pi (LPMP) + Vig dynamics. **(A)** Evaluation of the number of neurons created by the LPMP + Vig model over 100 m according to the vigilance threshold and the environment. **(B)** Illustration of the activities of the spatial neurons of the LPMP model during a navigation sequence. *Each vertical gray line* corresponds to the index of the frame at which the learning of a spatial neuron occurs. The *horizontal lines* correspond to an estimate of the space encoded by neuron. The first graph has a vigilance of 0.25 and the second of 0.15. **(C)** Evaluation of the average size of spaces encoded by LPMP spatial neurons as a function of the vigilance. The *dotted lines* represent the standard deviation around the mean value.

The novelty detector proposed in LPMP + Vig is controlled by *v*, the vigilance threshold. Indeed, this value controls the sizes of the place fields, as illustrated in the two graphs of Figure 9A: to encode a trajectory of a given length, the higher the vigilance, the more the number of neurons used increases in order to maintain a sufficient level of recognition. This increase in the number of neurons also decreases the average space encoded by a neuron on a sequence.

Figure 9A presents the average number of neurons generated over 100 m in the four types of environments as a function of the vigilance thresholds used. It shows that the *forest*, *city center*, and *suburb* environments required more neurons on average to maintain an equal level of recognition. Covering a trajectory of 100 m in the *forest* environment required 20 neurons more than for the *boulevard* and 5 neurons more than for the *city center* and the *suburb* environments.

Figure 9C completes this analysis by showing the average size and standard deviation of the spaces encoded by neurons per environment (place field plotted in dotted lines). It shows two phenomena: firstly, for the *boulevard* environment, the mean spaces of neurons were on average larger than in the *city center*, *forest*, and *suburb* environments, consistent with Figure 9A. Secondly, the standard deviation was much greater on *boulevard* than that on other environments, indicating that the LPMP + Vig model better generalized^8^ what the system has learned, thanks to a sufficient number of distal landmarks in the field of vision. The results are consistent with the performance results, which showed that the LPMP + Vig model gave better results on *boulevard* than on *suburb*.

### 6.4 Evaluation of Computational Cost

**FIGURE 10:**
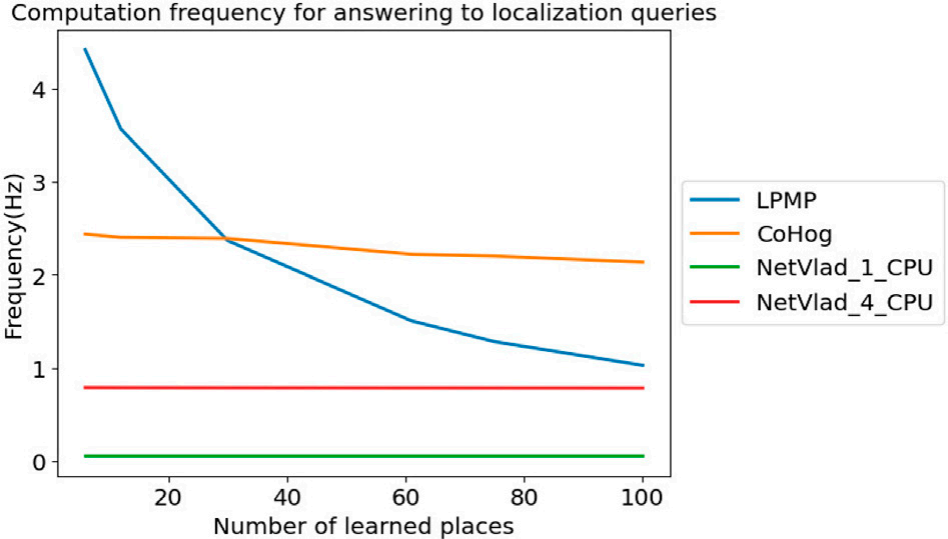
Frequency achieved for answering localization queries. This graph presents the frequency at which Log-Polar Max-Pi (LPMP), CoHog, and NetVLAD answered localization queries depending on the number of locations learned at an equal place sampling rate on the *boulevard* route.

Figure 10 shows the average frequency at which LPMP, LPMP + Vig, CoHog, and NetVLAD answered to localization queries, depending on the number of learned places. The graph shows that the LPMP model was the fastest when the number of locations is lower than 30, and then quickly slowed down until it almost reached the performance of the NetVLAD model using 4 CPUs. However, it was still superior to NetVLAD models on a single CPU, running at an average frequency of 0.05 Hz.

The dynamics of graph 10 could be explained by the memory cost of place coding and by the use of a log-polar transform to encode the visual information. Firstly, in the current version of the proposed model, the visual landmarks are systematically stored, without a process of merging the nearby landmarks. The memory used by LPMP thus becomes rather quickly large, slowing down the computation time. It should be noted that the model was still faster than NetVLAD in computation time, which used 4 CPUs.

Secondly, the log-polar transform was very fast to compute, unlike the encoding method proposed by NetVLAD and CoHog that took more time and computational resources. Thus, for small memory requests, the LPMP model is naturally faster on a single CPU. The log-polar, however, is primarily intended to robustify the system against small appearance changes induced by the movement of the vehicle, but is not intended to perform efficient compression of visual information. Thus, the code of the landmarks quickly becomes very large and requires a longer computation time, which takes over the fast encoding time.

## 7 Discussion

In this paper, the localization performance of the LPMP model, a bio-inspired neural network originally designed to study animal navigation (Gaussier and Zrehen, 1995), was assessed in a road environment. Far from the usually small and controlled environments used to reproduce experiments carried out in animals, this work highlighted the interest of such a neuromimetic approach when it is applied to road environments, characterized by much larger and more dynamic (human activities) environments. The model was thus evaluated in terms of localization performance and computing time, in cross-validation on a selection of sequences from the Oxford car dataset, presenting four different environments with various levels of urban activity.

Firstly, regarding the localization performance, the results showed that the LPMP model was more efficient than its direct competitor, CoHog, one of the best unsupervised models available in the literature (Zaffar et al., 2020a). It gave better answers on most environments and for most place sampling rates, whatever the presence of human activity in the dataset. Moreover, although it does not require any training, LPMP also gave competitive results with NetVLAD, which is one of the top-performing CNN models.

These results showed the interest of the one-shot learning mechanism and of the use of the spatial position of the landmark in the field of VPR. The one-shot learning mechanism allows the model to learn an accurate representation of a new location in a single iteration, allowing the system to achieve high localization scores. Moreover, the use of the spatial position of the landmark improves the performance of the model, at a low cost, and could benefit other algorithms. It should be noted that this information can be extracted from the images using a visual compass (Delarboulas et al., 2014), instead of a magnetic one.

For the LPMP + Vig model, the results showed that the use of a novelty detector gave much better results with high place sampling rates than the other models, highlighting the interest of a vigilance system for visual navigation. The use of a vigilance loop at small scales would make it possible to have less confusion between the codes of places because they are not created based on sampling, but based on the overall recognition of the system. However, the performance of LPMP + Vig deteriorated from a place sampling rate greater than 5 m. This degradation is explained by the size of the place fields of neurons that, above 5 m, may be too large to ensure perfect localization. A solution to this problem would be to adapt the learned representation during loop closure to adjust the stored place.

The experiments also revealed several important properties inherent in the use of a novelty detector mechanism. Thus, as stated in previous studies (Cuperlier, 2007; Espada et al., 2019), the mean size of the place field generated depends on two parameters: the value of the vigilance threshold and the type of environment. This variation in the cell dynamics shows that some environments are more difficult to process and require more neurons to maintain a given level of recognition. A solution could be to adapt an online the vigilance value according to the type of environment in order to obtain the best possible recognition performance.

Secondly, regarding the frequency achieved by the LPMP model, experiments have shown that the performance of the model decreased with the number of locations learned in the model. Two main reasons can explain this phenomenon: the growing number of neurons needed to code the landmarks and the size of the log-polar code used in the model.

For the first point, a major drawback of the model in its current version is that it systematically recruits new neurons to learn the visual signatures of landmarks when a new place is learned, regardless of the fact that very close signatures might have already been learned before. As a result, the system creates more landmarks than necessary, reducing the computation frequency of the LPMP model. Several solutions are therefore possible, for example the desynchronization of the learning signals of neurons coding places from those learning the landmark signatures, allowing to decrease the number of landmarks learned. In fact, they would be learned only when required, i.e., if no neuron already codes for the signature, instead of forcing the learning of the current signature as soon as a new neuron must code for a new place.

For the second one, the model in the proposed version does not compress the visual information before storing it in the landmark memory. The landmark code is quite large (54 × 54) and increases the computation time of the landmark memory. Thus, works have been undertaken to develop a sparse model of visual information representation (called HSD + MP) in order to improve the performance of the LPMP model while reducing its computational cost (Colomer et al., 2021).

The computational performance is, however, to be put in parallel with the use of the model, which adapts quite well to the SLAM architecture. Indeed, in numerous models, the localization is divided into two cases: the global localization on the map, in the case of loss of localization, and the local localization knowing the last position (Mur-Artal et al., 2015). Thus, a model such as LPMP, which is very fast to run on a reduced number of places but which gives better performance on high accuracies, seems to be very appropriate in this kind of case.

Moreover, from the implementation point of view, it should be noted that the experiments realized with the LPMP model were based on a lightly optimized software implementation. A gain remains to be envisaged by improving the software implementation, especially by using a code as optimized as CoHog or NetVlad. For example, switching to a parallelized code would thus maintain the performance of the LPMP model for a larger number of places. A particularly advanced hardware implementation, more suited to bio-inspired neural architecture, is being studied on a heterogeneous hardware solution in parallel to the current work (Elouaret et al., 2019).

Finally, from a neurobiological perspective, the spatial neurons generated by this model exhibited activities with properties closer to the *spatial view* cells than the *place* cells. This is coherent with our previous findings for indoor navigation (Gaussier et al., 2002) and confirms in outdoor environments the hypothesis that the difference may be due to the size (width) of the FOV. The same model, when used in a robotic setup with a fixed camera, results in spatial view cells, whereas when the camera can grab a panorama, the model generates cells closer to the omnidirectional place field exhibited by place cells.

## Data Availability

Publicly available datasets were analyzed in this study. These data can be found here: https://robotcar-dataset.robots.ox.ac.uk/.

## Footnotes

1 Information that might be obtained from a magnetic or visual compass (Giovannangeli and Gaussier, 2007).

2 The learning of a new place in the WM^p^ is conditioned by the learning of all the landmarks that compose it.

3 This information could be derived from a magnetic compass on a real vehicle or from the GPS coordinates provided in the datasets.

4 Null weights are used for the sake of simplicity and to increase the computing speed.

5 This property is especially relevant in monotonous environments such as a highway.

6 RobotCar Seasons (Sattler et al., 2018) proposes some sequences for VPR tests across seasons.

7 This proportion has been experimentally verified as being sufficient to give significant results.

8 Neurons that encode spaces of more variable sizes indicate that the system is able to adapt to the environment.
